# VR‐based Gamma Sensory Stimulation: A feasibility study

**DOI:** 10.1002/alz70859_106544

**Published:** 2025-12-25

**Authors:** Carolina Reis

**Affiliations:** ^1^ Clarity, Paris, Île‐de‐France France

## Abstract

**Background:**

Alzheimer's disease (AD) presents a critical global health challenge, with current therapies offering limited efficacy in halting disease progression. Gamma sensory stimulation (GSS) has emerged as a promising non‐invasive neuromodulation technique that enhances gamma neural synchrony, potentially counteracting AD pathology by reducing neuroinflammation, promoting glymphatic clearance, and improving synaptic plasticity. However, existing GSS delivery methods rely on simplistic sensory stimuli that lack user engagement, creating adherence barriers and limiting the full therapeutic potential of GSS.

**Method:**

This pilot study assessed the feasibility, safety, and efficacy of delivering GSS via virtual reality (VR), combining EEG recordings to monitor neural activity and a digital questionnaire to evaluate safety and tolerability. Sixteen cognitively healthy older adults participated in three experiments designed to assess modulation of gamma activity in response to 40Hz stimuli.

**Result:**

EEG recordings confirmed that unimodal auditory and visual stimuli reliably induced gamma oscillations in their respective cortical regions, while multimodal stimulation enhanced both gamma power and inter‐trial phase coherence, demonstrating robust modulation of gamma activity. Notably, significant gamma activity modulation was also achieved when diversified multimedia content was modulated at 40Hz and integrated into a cognitive task, highlighting the flexibility and effectiveness of this approach.

**Conclusion:**

The findings of this study validate VR as a scalable tool for delivering engaging and cognitively relevant GSS, paving the way for personalized therapies that maximize adherence and therapeutic outcomes. By integrating interactive elements, VR‐based GSS may uniquely target memory‐related neural networks, offering a novel approach to mitigate neurotoxicity and cognitive decline in AD.

